# Early Versus Late Contact with the Youth Justice System: Differences in Characteristics Measured at Birth, Child Protection System Contact and Adolescent Mental Health Outcomes.

**DOI:** 10.23889/ijpds.v7i3.1968

**Published:** 2022-08-25

**Authors:** Catia Malvaso, Michaela Magann, Pedro Malvaso, Alicia Montgomerie, Rhiannon Pilkington, Paul Delfabbro, John Lynch

**Affiliations:** 1 University of Adelaide

## Objectives

Although opioid prescribing and harms have increased in Australia, there is a lack of population-level evidence about the drivers of long-term opioid use, dependence and other harms. This study aims to profile the POPPY II cohort, with respect to sociodemographic and clinical health characteristics and patterns of opioid initiation.

## Approach

Data were from the Better Evidence Better Outcomes Linked Data (BEBOLD) platform including children in South Australia born 1991-2002, followed from birth to age 18 (n=249,995). Young people were categorised into three groups: 1) those who had their first YJ supervision before age 14, i.e., those who had ‘early’ contact; 2) those who had their first YJ supervision at age 14 or older, i.e., those who had ‘late’ contact; and 3) those who had no contact with the YJ system by age 18.

## Results

Of the 249,995 children born 1991-2002, 4,097 (1.6%) had YJ contact. Of these, 667 (16.3%) had their first YJ supervision early, and 3,430 (83.7%) had their first YJ supervision late. Compared to the late contact group, young people with early contact had more serious YJ contact patterns (e.g., 91% versus 59% ever experienced custodial supervision). Compared to the late contact and no YJ contact groups, the early contact group were: more disadvantaged at birth; had more serious child protection contact by age 10; and a higher proportion had experienced at least one mental health-related hospitalisation from ages 12-18.

## Conclusion

This analysis demonstrates the complex circumstances that precede and co-occur with YJ involvement. Early life adversity and poor adolescent mental health were more pronounced for young people who had early contact with YJ, compared to those who had late contact. This points to the need for investment in early supports.

